# Similar localization of conformational IgE epitopes on the house dust mite allergens Der p 5 and Der p 21 despite limited IgE cross‐reactivity

**DOI:** 10.1111/all.13398

**Published:** 2018-02-21

**Authors:** M. Curin, T. Garmatiuk, Y. Resch‐Marat, K. W. Chen, G. Hofer, K. Fauland, W. Keller, W. Hemmer, S. Vrtala, M. Focke‐Tejkl, R. Valenta

**Affiliations:** ^1^ Division of Immunopathology Department of Pathophysiology and Allergy Research Center for Pathophysiology, Infectiology and Immunology Medical University of Vienna Vienna Austria; ^2^ Institute of Molecular Biosciences BioTechMed University of Graz Graz Austria; ^3^ FAZ ‐ Floridsdorf Allergy Center Vienna Austria

**Keywords:** allergen, allergy, cross‐reactivity, house dust mite allergy, IgE epitope mapping

## Abstract

**Background:**

Due to high IgE recognition frequency and high allergenic activity, Der p 5 and Der p 21 are clinically important house dust mite (HDM) allergens. The objective of this study was to characterize the immunodominant IgE epitopes of Der p 5 and Der p 21 responsible for their high allergenic activity.

**Methods:**

A panel of 12 overlapping peptides spanning the Der p 5 and Der p 21 sequence were synthesized to search for sequential IgE epitopes by direct testing for allergic patients' IgE reactivity. Peptide‐specific antibodies raised in rabbits were used in inhibition studies for localizing conformational IgE epitopes which were visualized on the surfaces of the allergen structures by molecular modelling. IgE cross‐reactivity between the allergens was investigated by IgE inhibition studies.

**Results:**

Immunodominant IgE epitopes defined by allergic patients' IgE on Der p 5 and Der p 21 were primarily of the conformational, discontinuous type including N‐ and C‐terminal portions of the protein. They could be located on each allergen on one area with similar localization, but despite similar structure of the allergens, no relevant IgE cross‐reactivity could be detected.

**Conclusion:**

Our study shows that Der p 5 and Der p 21 contain a major conformational IgE epitope‐containing area located on similar portions of their structure, but they lack relevant IgE cross‐reactivity. These data are important for the development of modern allergy vaccines based on defined molecules for allergen‐specific immunotherapy of HDM allergy.

## 1 INTRODUCTION

House dust mites (HDMs) belong to the most potent and frequent allergen sources worldwide with a prevalence of sensitization of more than 40% within atopic populations.^1^, ^2^ House dust mite ‐sensitized patients suffer from a variety of allergic symptoms, in particular from respiratory and skin allergy, and often exhibit severe respiratory symptoms such as asthma.^3^, ^4^ Although HDM is a complex allergen source with more than 20 different reported allergen groups, only certain allergens are of high clinical relevance, among them the major allergens, Der p 1, Der p 2 and Der p 23 and a group of mid‐tier allergens including Der p 5 and Der p 21 which in some population are recognized by more than 50% of HDM‐allergic patients.^5^, ^6^, ^7^ Groups 5 and 21 house dust mite (HDM) allergens were identified in *Dermatophagoides pteronyssinus, Dermatophagoides farinae* and *Blomia tropicalis* and were shown in several studies to be important allergens with high allergenic activity in terms of inducing effector cell activation and skin inflammation.^8^, ^9^, ^10^, ^11^, ^12^ A recently published study investigating the prevalence of IgE recognition and the development of the evolution of IgE sensitizations to HDM allergens in a large birth cohort in Germany highlighted the importance of Der p 5 and Der p 21 because approximately 20% to 30% of the participants were sensitized against these allergens.^13^ In another study performed in HDM‐allergic children suffering only from rhinitis or rhinitis and asthma, sensitization to Der p 5 and Der p 21 occurred more frequently in the children who also suffered from asthma and asthmatic children more frequently recognized several different HDM allergens including Der p 5 and Der p 21 compared to children without asthma.^14^ Der p 5 and Der p 21 are therefore clinically important allergens, but it has been found that these two allergens are poorly represented in currently manufactured allergen extracts.^15^ It is therefore difficult to develop HDM allergy vaccines based on natural allergen extracts which protect HDM‐allergic patients who are sensitized to these allergens. One possibility to overcome the limitations of natural allergen extracts for HDM‐specific immunotherapy is therefore to develop molecular vaccines which are based on recombinant allergens, allergen derivatives or allergen‐derived peptides.^16^ Several new forms of allergy vaccines have been successfully evaluated in clinical trials which are based on defined molecular components.^17^, ^18^ The construction of these vaccines depends heavily on the definition of the clinically relevant allergens of a given allergen source and the knowledge of IgE and T‐cell epitopes of the key allergens. In fact, the three‐dimensional structure of Der p 5 has been revealed and shows a three‐helical bundle that can polymerize to create a hydrophobic cavity which could possibly be a ligand‐binding site.^19^ Also Der p 21 seems to have a similar three‐dimensional structure (ie, forms a three‐helical bundle protein) but shows no IgE cross‐reactivity with Der p 5.^10^ Here, we performed a detailed analysis of the IgE epitopes of Der p 5 and Der p 21 to break the ground for a broadly protective HDM allergy vaccine.

## 2 MATERIALS AND METHODS

### 2.1 Synthetic peptides, rDer p 5 and rDer p 21, molecular modelling

Peptides were synthesized using Applied Biosystems peptide synthesizer Model 433A (Foster City, CA, USA) and purified by HPLC as described previously.^20^ Identity of each of the synthetic peptides was confirmed by matrix‐assisted laser desorption ionization‐time of flight (MALDI‐TOF) analysis (Bruker, Billerica, MA, USA). For the purpose of rabbit immunization, each of the peptides was coupled to keyhole limpet haemocyanin (KLH) (MW 4.5 × 10^5^−1.3 × 10^7^ Daltons; Pierce, ThermoFisher Scientific, Waltham, MA, USA) and purified using a conjugation kit according to manufacturer's instructions (Pierce, ThermoFisher Scientific). Table S1 summarizes the position, length and biochemical properties of the Der p 5‐ and Der p 21‐derived synthetic peptides. Recombinant (r) Der p 5 and Der p 21 were expressed in *E. coli* and purified as described elsewhere.^9^, ^10^ A model of the Der p 5 structure was created based on the NMR structure of Blo t 5 deposited in the protein data bank (http://www.rcsb.org/pdb/home/home.do; PDB: 2JMH) because it represented a monomeric solution structure.^21^ For modelling of Der p 21, the NMR structure of Blo t 21 was used (PDB: 2LM9). Both models were made using the program SWISS‐MODEL.^22^ The solvent‐accessible surface (SAS) of each amino acid residue was calculated with the program MSMS.^23^ SAS is determined by a spheric solvent probe (r = 1.4 Å) rolling over the van der Waals surface of the protein and is displayed in Å^2^. The surface exposure of the peptides was calculated as follows: surface exposure [%] = SAS of all amino acids of the peptide/SAS of all amino acids of the protein*100. Graphic depictions of the model were rendered with PyMOL (PyMOL Molecular Graphics System, version 1.7.4; Schrodinger, NY).

### 2.2 ELISA competition assay for analysing the inhibition of human IgE binding to rDer p 5 or rDer p 21 with peptide‐specific antibodies

ELISA plates were coated overnight with 1 μg/mL rDer p 5 or rDer p 21, blocked for 2.5 hours and were then pre‐incubated for 24 hours with anti‐Der p 5 peptide antisera, mix of anti‐Der p 5 peptide antisera, anti‐Der p 5 antiserum, anti‐Der p 21 peptide antisera, mix of anti‐Der p 21 peptide antisera, anti‐Der p 21 antiserum, or, for control purposes, with the corresponding pre‐immune sera. As each peptide was used for immunization of 2 rabbits with CFA and 2 rabbits with Al(OH)_3_, antisera derived from two rabbits were combined in ratio 1:1 to a total sera dilution of 1:50 for both, CFA‐immunized rabbits and Al(OH)_3_‐immunized rabbits. After overnight incubation with sera from HDM‐allergic patients (diluted 1:5), bound IgE antibodies were detected with the horseradish peroxidase‐labelled goat anti‐human IgE antibodies (KPL, Gaithersburg, MD, USA). The percentage reduction in IgE binding achieved by means of pre‐incubation with rabbit antisera was calculated as follows: 100‐(OD_I_/OD_P_) × 100), where OD_I_ and OD_P_ represent optical density values after pre‐incubation with the rabbit immune serum or pre‐immune serum, respectively. Cross‐inhibition experiments were performed as described above, rDer p 5 and rDer p 21 were coated on ELISA plates, and after blocking, plates were pre‐incubated with anti‐Der p 5 antiserum, anti‐Der p 21 antiserum, anti‐Der p 5‐peptide antisera or anti‐Der p 21‐peptide antisera. ELISA plates were then incubated with patients’ sera (diluted 1:5) which were double positive to Der p 5 and Der p 21 to test for cross‐reactive epitopes.

## 3 RESULTS

### 3.1 Characterization of synthetic peptides spanning the Der p 5 and Der p 21 sequence and their localization

**Figure 1:**
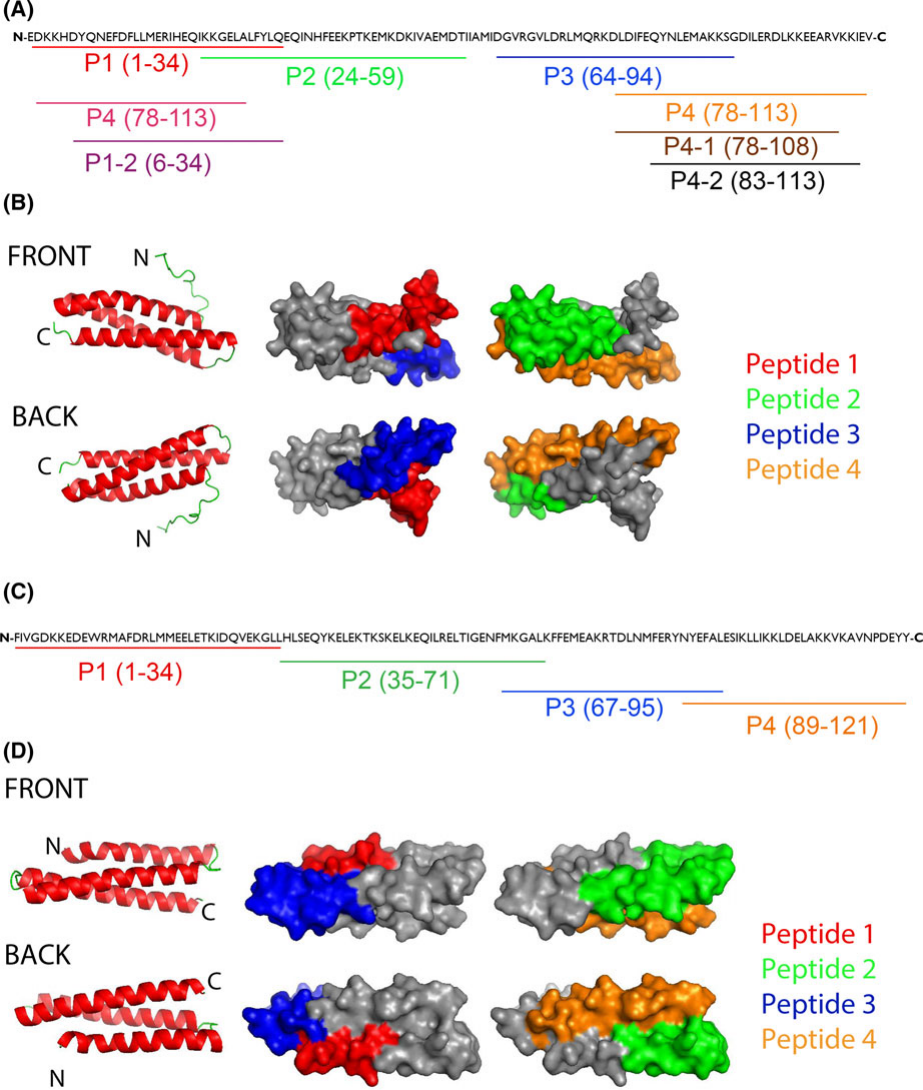
Der p 5‐ and Der p 21‐derived peptides. A, The position of the peptides is indicated in the Der p 5 amino acid sequence (P1‐4). Shortened peptides P1‐1, P1‐2, P4‐1, P4‐2 are also shown. B, Localization of the peptides (P1‐4) in a model of the three‐dimensional structure of Der p 5. Left images: ribbon representation of the Der p 5 structure (view on the front and back). Central and right images: corresponding surface representations of the Der p 5 structure with the peptides highlighted in different colours (P1, red; P2, green; P3, blue; P4, orange). C, The position of the peptides is indicated in the Der p 21 amino acid sequence (P1‐4). D, Localization of the peptides in a model of the three‐dimensional structure of Der p 21. Left images: ribbon representation of the Der p 21 structure (view on the front and back). Central and right images: corresponding surface representations of the Der p 21 structure with the peptides highlighted in different colours (P1, red; P2, green; P3, blue; P4, orange)

In a first approach, we covered the Der p 5 sequence with 4 peptides (P1‐P4) having a length from 32 to 37 amino acids (Figure 1A, Table S1). Figure 1B shows the localization of the four peptides on the three‐dimensional structure of Der p 5. In the given view, P1 and P2 appear on the front side, whereas P3 and P4 are located on the backside of the modelled structure (Figure 1B). The coverage of surface by the individual Der p 5 peptides was as follows: peptide 1: 34%, peptide 2: 28%, peptide 3: 29% and peptide 4: 31%.

Similar as for Der p 5, we were able to cover the sequence of Der p 21 with four overlapping peptides (P1‐ P4) with a length from 29 to 36 amino acids (Figure 1C, Table S1). When the Der p 21‐derived peptides 1‐4 were projected on the 3D model of the Der p 21, P1 and P3 appeared to define a surface patch close to the N‐terminus, while P2 and P4 define a patch on the opposite side of the molecule where the C‐terminus is located (Figure 1D). The coverage of surface by the Der p 21 peptides was as follows: Peptide 1: 14.3% (only aa 21‐34 could be calculated), peptide 2: 36.8%, peptide 3: 31.5% and peptide 4: 26.5%. Table S1 summarizes the amino acid sequence, position, length and biochemical properties of Der p 5‐ and Der p 21‐derived synthetic peptides. Each of the peptides was soluble in physiological buffers and lacked secondary structure as determined by circular dichroism measurements (data not shown) whereas both recombinant allergens were folded.^9^, ^10^

### 3.2 The majority of HDM‐allergic patients show IgE reactivity only to complete folded Der p 5 and Der p 21 but not to the unfolded peptides

**Figure 2:**
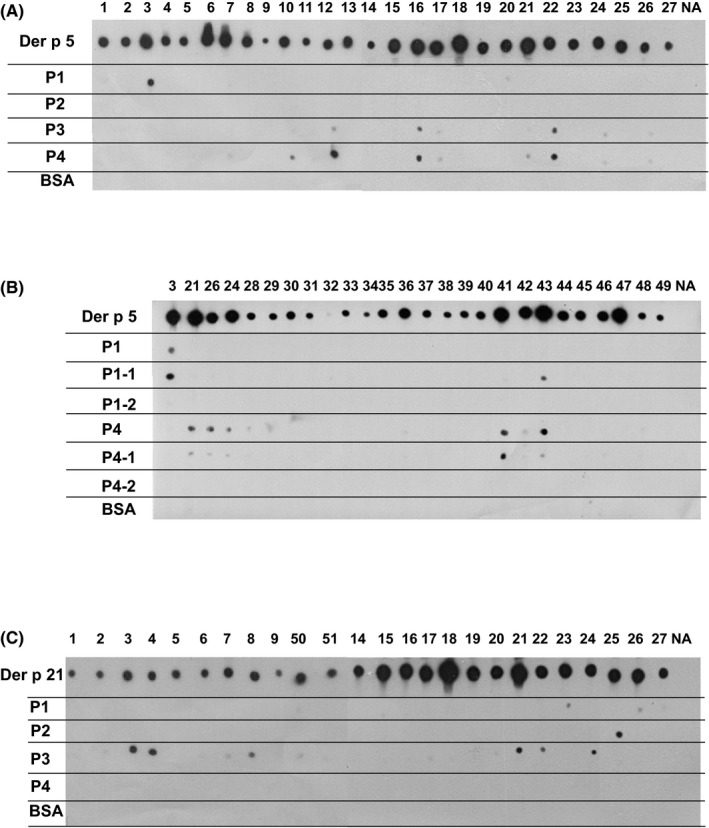
A, IgE reactivity of Der p 5 and Der p 5 peptides. Nitrocellulose dotted Der p 5, Der p 5‐derived peptides(P1‐P4) and BSA were tested for IgE reactivity with sera form 27 house dust mite (HDM)‐allergic patients 1, 2, 3, 4, 5, 6, 7, 8, 9, 10, 11, 12, 13, 14, 15, 16, 17, 18, 19, 20, 21, 22, 23, 24, 25, 26, 27 and a serum from a nonallergic individual (NA). B, Shortened Der p 5 peptides 1 and 4 lack IgE reactivity. Nitrocellulose‐dotted Der p 5, Der p 5 peptides P1 and P4 as well as N‐ or C‐terminally shortened P1 and P4 derivatives (P1‐1, P1‐2, P4‐1 and P4‐2) and BSA were tested for IgE reactivity with sera form 26 HDM ‐allergic patients (3‐49) and a serum from a nonallergic individual (NA). C, IgE reactivity of Der p 21 and Der p 21 peptides. Nitrocellulose‐dotted Der p 21, Der p 21‐derived peptides (P1‐P4) and BSA were tested for IgE reactivity with sera form 25 HDM ‐allergic patients (1‐51) and a serum from a nonallergic individual (NA). Bound IgE was detected with ^125^I‐labelled anti‐human IgE antibodies and visualized by autoradiography

The IgE‐binding capacity of the four Der p 5‐derived peptides was compared with complete rDer p 5 allergen in a nondenaturing RAST‐based IgE‐binding dot‐blot assay using sera from 27 Der p 5‐positive HDM‐allergic patients (Figure 2A, lanes 1‐27). Each of the patients showed IgE reactivity to rDer p 5, whereas the peptides showed no (ie, P2) or weak (ie, P1, P3, P4) IgE reactivity with few sera (Figure 2A) indicating that patients mainly recognize the folded Der p 5 molecule. P3 and P4 seemed to contain minor overlapping sequential IgE epitopes which were recognized by the same patients (ie, patients 12, 16, 17, 22, 24 and 26) (Figure 2A). Serum from a nonallergic individual (NA) showed no IgE reactivity to rDer p 5 or to Der p 5‐derived peptides (Figure 2A, lane NA), and none of the patients showed IgE reactivity to BSA (Figure 2A).

To further reduce the IgE reactivity of the peptides, we shortened the length of P1 and P4 for 5 amino acids at their C‐ (P1‐1, P4‐1) or N‐termini (P1‐2, P4‐2) (Figure 1A, Table S1). Shortening of the peptides at their C termini did not abolish patients IgE binding to P1‐1 and P4‐1 completely but when the peptides were shortened at the N‐termini, IgE binding to P1‐2 and P4‐2 was completely abolished (Figure 2B, patients 3‐49).

IgE reactivity of the four Der p 21‐derived peptides was compared with that of complete Der p 21 in 25 Der p 21‐sensitized patients by dot‐blot assay (Figure 2C). P4 showed no IgE reactivity, P1 showed weak, barely detectable IgE reactivity with two sera, P2 reacted weakly with one serum, and P3 showed residual IgE reactivity with 10 of the patients’ sera (Figure 2C). Again no reactivity was observed with serum from a nonallergic subject, and the control protein BSA also showed no IgE reactivity (Figure 2C).

### 3.3 Immunization with non‐IgE‐reactive Der p 5‐ and Der p 21‐derived peptides induces allergen‐specific IgG antibodies

In a first set of experiments, we show that immunization of rabbits with each of the four KLH‐coupled Der p 5‐derived peptides (P1, P2, P3 and P4) induced IgG antibodies that recognized Der p 5 at dilutions of the antisera between 1:1.000‐1:5.000 with P4 being the most immunogenic peptide (Figure S1A‐D). A previously described anti‐Der p 5 antiserum raised with rDer p 5 and Freund's adjuvant^9^ was used for comparison and showed higher titres than the peptide‐specific antisera (Figure S1A‐D).

In a second set of experiments, we immunized rabbits with shortened versions of P1 and P4 (ie, P1‐2 and P4‐2) to compare the immunogenicity of the shortened peptides with the longer versions. In these immunizations, we included also a rabbit which was immunized with alum‐adsorbed complete rDer p 5. Figure S2A,B shows the IgG reactivity of the antisera obtained by immunization with the shortened peptides demonstrating that they induced comparable anti‐Der p 5 antibody titres as those obtained with the long peptides and with complete rDer p 5 adsorbed to alum.

Likewise, we compared the allergen‐specific antibody titres of rabbits which had been immunized with KLH‐coupled Der p 21‐derived peptides with those of rabbits immunized with the complete rDer p 21 (Figure S3). We found that peptides P1, P2 and P4 induced almost comparable levels of Der p 21‐specific antibodies as rDer p 21 (Figure S3A,B,D). Only P3 induced lower Der p 21‐specific antibodies (Figure S3C).

### 3.4 IgE inhibition experiments indicate that Der p 5 and Der p 21 contain a conformational IgE epitope area constituted by their N‐ and C‐terminal portions

The lack of relevant IgE reactivity against the Der p 5‐derived peptides in patients that reacted with intact, folded Der p 5 suggested that the major IgE epitopes of Der p 5 are likely discontinuous and/or conformational epitopes. It is possible to map the localization of such epitopes with the use of peptide‐specific antibodies that can recognize the folded complete antigen by competitive binding ELISA experiment.^24^ In these ELISA competition experiments, peptide‐specific antibodies are used to block allergic patients IgE binding and the degree of blocking obtained can be expressed as percentage of inhibition of IgE binding. Table 1 shows the percentages of inhibition of HDM‐allergic patients’ (n = 13) IgE binding obtained by pre‐incubation of Der p 5 with antisera against the individual peptides (P1‐P4), for a Der p 5‐specific antiserum and for a mix of anti‐P1 and anti‐P4 antibodies.

**Table 1:** Inhibition of patients' IgE binding to Der p 5 with antisera specific for Der p 5 and Der p 5 peptides

Patient	Inhibition %					
	Anti‐Der p 5	Anti‐P1	Anti‐P2	Anti‐P3	Anti‐P4	Anti‐P1+4
Alum						
1	82	40	35	32	44	56
8	95	31	25	30	38	50
10	92	27	27	28	37	63
11	65	22	16	16	31	53
22	87	22	22	31	36	44
35	68	23	4	21	28	60
39	53	27	6	7	31	40
41	86	46	31	47	57	76
42	80	55	34	53	62	76
47	74	41	19	21	30	59
52	88	39	28	36	58	64
53	75	47	34	47	60	78
54	82	33	16	29	40	62
Mean inh%	79	35	23	31	42	60
CFA						
1	80	63	36	70	86	84
8	92	62	41	61	87	83
10	92	45	30	79	94	90
11	73	24	19	58	87	82
22	88	38	30	65	85	81
35	73	53	34	66	71	72
39	60	38	27	47	55	51
41	88	65	53	79	88	88
42	81	72	57	79	79	80
47	77	53	33	60	77	77
52	88	56	33	77	90	85
53	81	64	45	79	91	78
54	82	57	38	73	83	80
Mean inh%	81	53	37	69	83	79

The percentages of inhibition of patients’ IgE binding to Der p 5 after preincubation with the individual antipeptide antibodies (anti‐P1, anti‐P2, anti‐P3 or anti‐P4), mix of anti‐P1+P4 or with anti‐Der p 5 antibodies obtained vs inhibition with pre‐immune sera are shown.

When alum was used as an adjuvant for immunization of the rabbits, the highest mean inhibition was obtained by anti‐P4 serum (ie, 42%) followed by anti‐P1 with a mean inhibition of 35% (Table 1). Somewhat lower inhibitions were obtained by anti‐P3 and anti‐P2 (31% and 23%). Combinations of anti‐P1 and anti‐P2 antibodies as well as of anti‐P3 and anti‐P4 antibodies did not give relevant increases in IgE inhibition (data not shown). However, when the mix of anti‐P1 and anti‐P4 antibodies was tested, the mean inhibition could be increased to 60% which was almost in the range of the inhibition obtained with antibodies raised against the complete Der p 5 allergen (79% mean inhibition).

With antisera induced by immunization with CFA stronger inhibitions were obtained because the titres of the antisera were higher than those of the antisera obtained with alum (Figure S1, S2, S3) (Table 1, bottom part). Here, the inhibition obtained by anti‐P4 antibodies was also the highest among the peptide antisera (mean inhibition of 83%) which was comparable with the inhibition obtained with rabbit antibodies raised by immunization with the complete Der p 5 allergen (mean inhibition 81%) (Table 1). Antibodies raised against P3 which overlaps for 17 amino acids with P4 also strongly inhibited IgE binding to Der p 5 (mean inhibition 69%). The mean inhibition with anti‐P1 was 53%, and the lowest mean inhibition was obtained for P2‐specific antibodies (37%). These results indicate that major IgE‐binding epitopes are present on the N‐terminus and C‐terminus of Der p 5 in the portions containing P1 and P4.

A further refinement of the IgE‐binding sites was obtained with IgE inhibition experiments using antibodies raised against shortened versions of P1 and P4 (ie, anti P1‐2 and anti‐P4‐2 antisera). Table S2 shows that, regardless whether CFA or alum was used as adjuvant, a combination of antisera against P1‐2 and P4‐2 inhibited IgE binding to Der p 5 as well as antibodies raised against complete Der p 5.

We then mapped Der p 21 IgE epitopes using anti‐Der p 21 peptide antibodies (Table 2). The strongest inhibition of patients’ IgE binding to Der p 21 was obtained with anti‐P4 antibodies, which caused a mean inhibition of IgE binding of 64% in the case of alum‐immunized rabbits and 81% for the CFA‐immunized rabbits and thus was comparable to the inhibition obtained with antibodies raised against the complete Der p 21 allergen (mean inhibition of 66% for alum and 68% for CFA). The second strongest inhibitor was the anti‐P1 antiserum with a mean inhibition of IgE binding of 54% for alum and 55% for CFA rabbits (Table 2). A low mean inhibition of IgE binding of 14% (alum) and 39% (CFA) was obtained with anti‐P2 antibodies, while almost no inhibition was obtained with anti‐P3 antibodies (<10%) (Table 2, data not shown). Again we noticed that a combination of anti‐P1 and anti‐P4 antibodies raised with alum inhibited IgE binding to Der p 21 best, whereas combinations of anti‐P1 and anti P2 or anti‐P2 and anti‐P4 antibodies were less effective (Table 2). These results indicate that P1 and P4 are part of a conformational IgE epitope‐containing area on Der p 21 which is constituted by the N‐terminal and C‐terminal portion of Der p 21.

**Table 2:** Inhibition of patients IgE binding to Der p 21 with antisera specific for Der p 21 and Der p 21 peptides

Patient	Inhibition %							
	Anti‐Der p 21	Anti‐P1	Anti‐P2	Anti‐P4	Anti‐P1 +2	Anti‐P1 +4	Anti‐P2+4	Anti‐P1 +2+4
Alum								
14	73	47	15	48	58	79	66	86
17	72	54	13	64	67	86	75	90
19	67	52	15	75	61	86	82	88
21	66	59	22	72	65	81	76	84
22	71	61	19	69	76	87	77	90
24	76	67	17	77	74	89	81	90
43	64	49	4	48	61	80	69	87
44	53	38	9	41	49	72	54	81
Mean inh%	66	54	14	64	64	83	73	87
CFA								
14	73	60	33	78	77	88	82	90
17	75	68	48	87	83	89	88	91
19	69	54	34	85	77	87	87	89
21	63	50	34	78	73	84	80	86
22	67	64	48	84	80	87	85	86
24	76	71	48	85	84	88	85	89
43	62	47	37	80	74	83	87	87
44	70	43	31	75	74	86	87	89
Mean inh%	68	55	39	81	77	86	85	88

The percentages of inhibition of patients’ IgE binding to Der p 21 after pre‐incubation with the individual antipeptide antibodies (anti‐Pi, anti‐P2 or anti‐P4), mixes of anti‐P1+2, anti‐P1+4, anti‐P2+4, anti P1+2+4 or with anti‐Der p 21 antibodies obtained vs inhibition with preimmune sera are shown.

**Figure 3:**
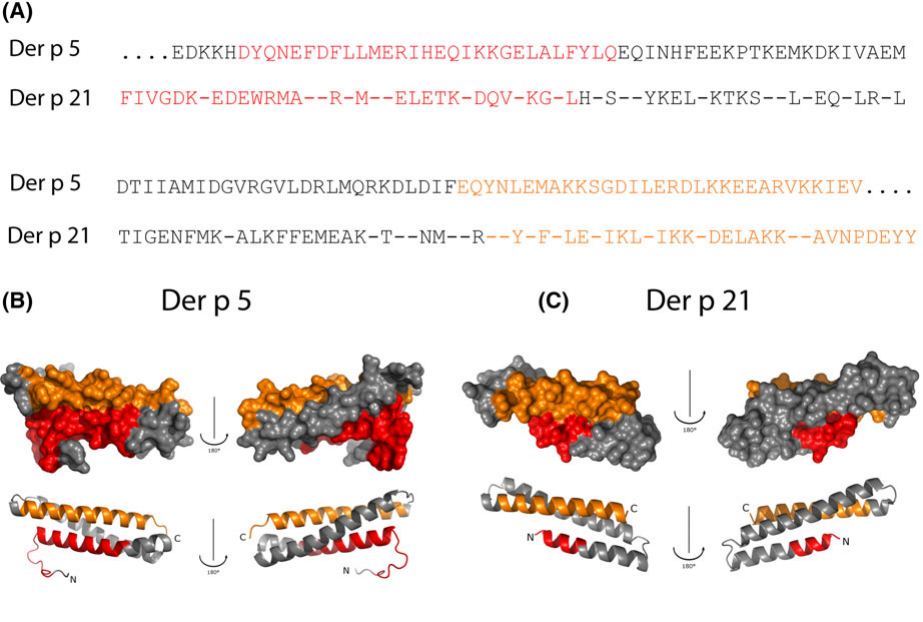
Localization of the areas on Der p 5 and Der p 21 containing conformational IgE epitopes. A, Position of the Der p 5 peptides (P1‐2, P4‐2) and the Der p 21 peptides (P1, P4) in the allergen sequences. Visualization of the areas containing the conformational IgE epitopes on a surface and ribbon representation of Der p 5 (B) and Der p 21 (C) (front sides: left; back sides; right)

Figure 3 presents a summary of the IgE epitope mapping obtained for Der p 5 and Der p 21. We have indicated the peptides defined by the antisera which inhibited IgE binding to Der p 5 (ie, P1‐2 and P4‐2) and Der p 21 (ie, P1 and P4) in the primary sequence of the allergens where they are part of the N‐ and C‐terminal portions of the allergens (Figure 3A). In the folded allergens, the portions defined by these peptides assemble conformational epitope‐containing areas on Der p 5 (Figure 3B) and Der p 21 (Figure 3C).

### 3.5 Der p 5 and Der p 21 show limited cross‐reactivity at IgG and IgE level despite similar structural distribution of IgE epitopes

Der p 5 and Der p 21 both have similar alpha helical structure and IgE epitopes are located in similar regions, but earlier IgE inhibition studies showed that there is no relevant cross‐reactivity of IgE antibodies between the two molecules.^10^ Nevertheless, we further investigated whether there might be cross‐reactive epitopes on the two allergens. When Der p 5, Der p 21, Der p 5‐ and Der p 21‐derived peptides were probed with rabbit IgG antibodies derived against the complete allergens and allergen‐derived peptides, some cross‐reactivity could be detected (Figure S4A). Anti‐Der p 5 rabbit IgG recognized Der p 21 and the Der p 21‐derived peptide P3 and anti‐Der p 21 rabbit IgG recognized Der p 5 and the Der p 5‐derived peptides P3, P4 and P4‐1. The anti‐Der p 5 P1 antiserum recognized Der p 21 P1, and Der p 21 P3 was recognized by anti‐Der p 5 P3 and anti‐Der p 5 P4. However, antibodies derived against the shortened version of Der p 5 P4 (ie, anti‐Der p 5 P4‐2) did not recognize Der p 21 P3 and allowed to narrow the cross‐reactive region down to an amino acid motif which is similar in Der p 5 and Der p 21 (ie, DLDIF Der p 5 and DLNMF in Der p 21) (Figure S4A,B). Antibodies specific for Der p 21 P3 recognized Der p 5 P4 and Der p 5 P4‐1 but not the N‐terminally shortened Der p 5 P4‐2 peptide which lacks the DLDIF sequence (Figure S4A,B). We then re‐investigated whether the cross‐reactivity between Der p 5 and Der p 21 is present at the IgE level using an approach which is different from the previously performed IgE inhibition studies.^10^ In fact, we used rabbit anti‐allergen and antipeptide antisera to inhibit IgE binding of patients who had IgE antibodies to both allergens (ie, Der p 5 and Der p 21) (n = 5). For these sera, a very limited IgE cross‐reactivity could be found (Table 3). The mean inhibition of patients’ IgE binding to Der p 5 by anti‐Der p 5 antibodies was 78% and only 12% with anti‐Der p 21 antibodies. Der p 21 peptide antibodies did not show any relevant inhibition of the binding of patients IgE to Der p 5 (Table 3). When Der p 21 was immobilized on ELISA plates, a mean inhibition of IgE binding of 81% was found with anti‐Der p 21 antibodies and again a very low inhibition was obtained with anti‐Der p 5 antibodies (ie, 20% mean inhibition) (Table 3). Anti‐Der p 5 P4 antibodies caused a mean inhibition of IgE binding to Der p 21 of 13% (vs 80% inhibition to Der p 5) and anti‐Der p 5 P3 antibodies inhibited 8% of IgE binding to Der p 21 compared to 60% inhibition of IgE binding to Der p 5 (Table 3).

**Table 3:** Inhibition of patients IgE binding to Der p 5 and Der p 21 with antisera specific for Der p 5, Der p 21, Der p 5‐ and Der p 21‐derived peptides

Patient	Inhibition %									
	Anti‐Der p 5	Anti‐Der p 21	Anti‐Der p 5P1	Anti‐Der p 21P1	Anti‐Der p 5 P2	Anti‐Der p 21 P2	Anti‐Der p 5 P3	Anti‐Der p 21 P3	Anti‐Der p 5 P4	Anti‐Der p 21 P4
Der p 5 coated										
56	65	6	39	4	33	3	57	−1	76	2
8	93	9	58	0	59	2	68	−4	90	−5
57	68	32	67	0	61	0	73	8	77	5
9	73	7	52	−3	19	3	50	−3	75	0
20	90	8	14	−2	11	−2	51	−5	80	−1
Mean inh%	78	12	46	0	37	1	60	−1	80	0
Der p 21 coated										
56	27	67	1	63	4	61	11	36	27	59
8	12	90	1	68	7	59	8	23	15	81
57	28	79	0	77	7	68	8	35	13	74
9	15	81	0	67	4	62	6	37	4	75
20	20	90	4	80	3	72	5	28	7	78
Mean inh%	20	81	1	71	5	64	8	32	13	73

The percentages of inhibition of patients’ IgE binding to Der p 5 and Der p 21, respectively, after pre‐incubation with the individual anti‐peptide antibodies or with anti‐Der p 5 or anti‐Der p 21 antibodies obtained vs inhibition with pre‐immune sera are shown. Mean values are shown in the bottom line.

## 4 DISCUSSION

House dust mites are one of the most potent and frequent respiratory allergen sources worldwide. Der p 5 and Der p 21 are important allergens in HDM which, depending on the population of patients, are recognized by 30%‐60% of HDM‐allergic patients.^5^, ^6^ Moreover, both Der p 5 and Der p 21 are highly potent and clinically relevant allergens as demonstrated in basophil activation assays for Der p 5 and Der p 21 and by skin testing for Der p 5.^8^ In fact, the results from basophil activation testing indicate that Der p 5 and Der p 21 can induce basophil degranulation in sensitized patients at lower concentrations than the major HDM allergens.^9^, ^10^ The three‐dimensional structure of Der p 5 consists of three antiparallel alpha helices, and Der p 21 seems to have a similar structure, but interestingly, there is no relevant IgE cross‐reactivity among the two allergens.^10^ In this study, we used sera from HDM‐allergic patients to study the nature and localization of the IgE epitopes on Der p 5 and Der p 21. For this purpose, we used two different technologies: first we synthesized peptides of at least 30 amino acids length to search for the presence of sequential epitopes, but only few allergic patients showed low IgE reactivity to the peptides. Thus, Der p 5 and Der p 21, similar as most of the potent respiratory allergens,^25^ seemed to contain conformational but not sequential IgE epitopes. To determine those areas on the Der p 5 and Der p 21 structures which are part of the conformational epitopes, we followed an indirect strategy for epitope mapping. We raised rabbit antisera against the allergen‐derived peptides and used these rabbit antisera to block the binding of allergic patients IgE to the allergens. The results of the IgE inhibition experiments identified for each of the two allergens major IgE epitope‐containing areas which were defined by two peptides, one from the N‐terminus and one from the C‐terminus of the allergen. Within the allergen structure, the peptides were located in close vicinity and therefore seemed to be part of a discontinuous conformational epitope‐containing area. Interestingly, the major IgE epitope‐containing area was located on similar portions of the Der p 5 and Der p 21 structures although there was no relevant IgE cross‐reactivity between the two allergens. In this context, it is noteworthy that the corresponding allergens, Blo t 5 and Blo t 21 from the tropical mite *Blomia tropicalis,* have a three‐dimensional structure which is similar to that of Der p 5 and Der p 21. IgE cross‐reactivity between Blo t 5 and Der p 5 has been reported,^26^ but there is only low cross‐reactivity between Blo t 5 and Blo t 21.^27^ Available data suggest that point mutations in the C‐terminal portion of Blo t 21 can reduce the binding of allergic patients IgE. Regarding Blo t 5, IgE epitopes have also been localized to the C‐terminal portion of the molecules by site‐directed mutagenesis and by identifying IgE‐reactive peptides.^28^ These data suggest that similar portions on the three‐dimensional structure of group 5 and group 21 allergens from HDMs and tropical mites are major targets for IgE antibodies of allergic patients. These IgE epitope‐containing areas seem to be highly potent in selecting B cell receptors (ie, paratopes) and in inducing IgE responses in subjects from different populations in different parts of the world. However, there seems to be selectivity for the group 5 and group 21 epitopes among the B cells of the individual patients which seems to be due to differences among the allergen sequences. The surprising overall conservation of the localization of IgE epitope‐containing areas in structurally similar allergens which exhibit low or no IgE cross‐reactivity is not only of basic immunological interest but is also important for the design of allergy vaccines. First of all, it will be important to include both group 5 and group 21 allergens into a broadly protective HDM allergy vaccine because there is little IgE cross‐reactivity between Der p 5 and Der p 21. In this context, we found that immunization of rabbits with Der p 5 and Der p 21 induced robust IgG responses against Der p 21 and Der p 5, respectively (Figure S4A). However, anti‐Der p 5 and anti‐Der p 21 antibodies did not inhibit binding of allergic patients IgE to Der p 21 and Der p 5, respectively, in a relevant manner (Table 3). Likewise, IgG antibodies raised against the peptides from the IgE epitope‐containing areas of Der p 5 and Der p 21 did not inhibit allergic patients IgE binding to Der p 21 and Der p 5, respectively (Table 3). Second and most important, our study has mapped the major IgE epitope‐containing areas of Der p 5 and Der p 21 and we could identify nonallergenic peptides from these areas which can be used for the construction of allergy vaccines based on carrier‐bound B cell epitopes.^18^, ^29^ A vaccine based on nonallergenic B cell epitope‐derived peptides of the four major timothy grass pollen allergens has in fact been described^30^ and was shown to be clinically effective in immunotherapy trials in grass pollen‐allergic patients.^31^, ^32^, ^33^ Our work therefore will be important for the rational engineering of a recombinant vaccine for the treatment of HDM allergy.

## CONFLICTS OF INTEREST

Rudolf Valenta has received research grants from Biomay AG, Vienna, Austria, and Viravaxx, Vienna, Austria. He is a consultant for Biomay AG and Viravaxx, Austria. Other authors declare that they have no conflict of interest.

## AUTHOR CONTRIBUTIONS

RV designed and coordinated the study, participated in the interpretation of the findings, wrote and critically revised the manuscript, read and revised the final version. MC contributed to design of the study, performed experiments, participated in the interpretation of the findings, wrote the manuscript and revised the final version. WH kindly provided sera of HDM‐allergic subjects, read and revised the manuscript. MFT, SV and WK contributed to design and planning of the study, interpretation of findings, read and revised the manuscript. TG, YRM, KWC, GH and KF performed experiments, read and revised the manuscript.
