# Supplementary figures and images for: Similar localization of conformational IgE epitopes on the house dust mite allergens Der p 5 and Der p 21 despite limited IgE cross‐reactivity
Source: Allergy. 2018 Feb 21;73(8):1653–61. doi: 10.1111/all.13398 (PMC6055609; doi:10.1111/all.13398)

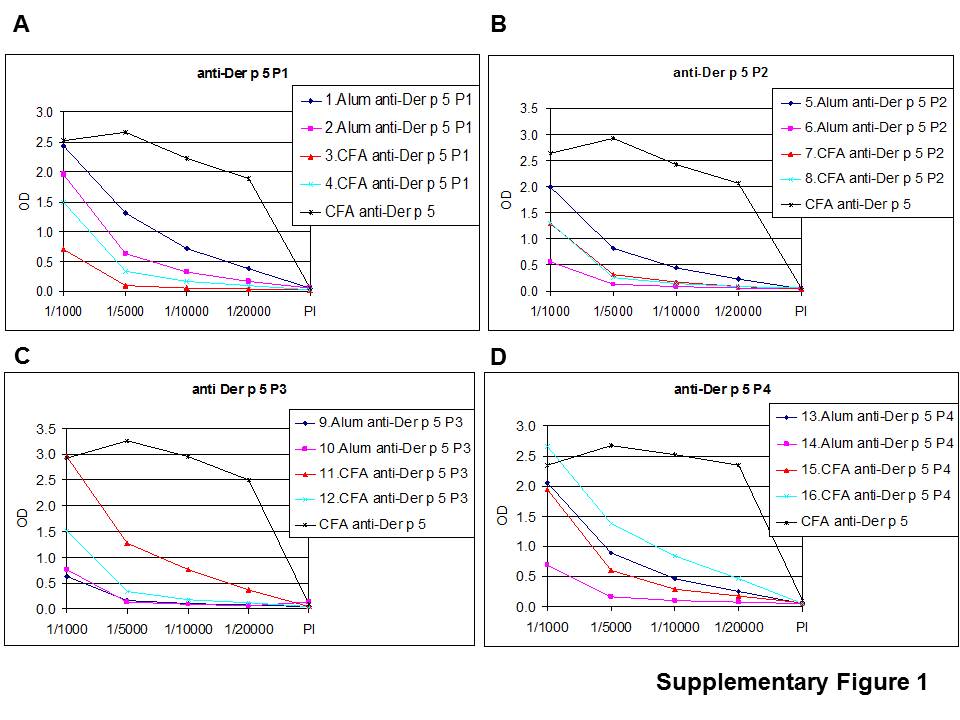

Supplement: Supplementary file 1 [file ALL-73-1653-s001.JPG]

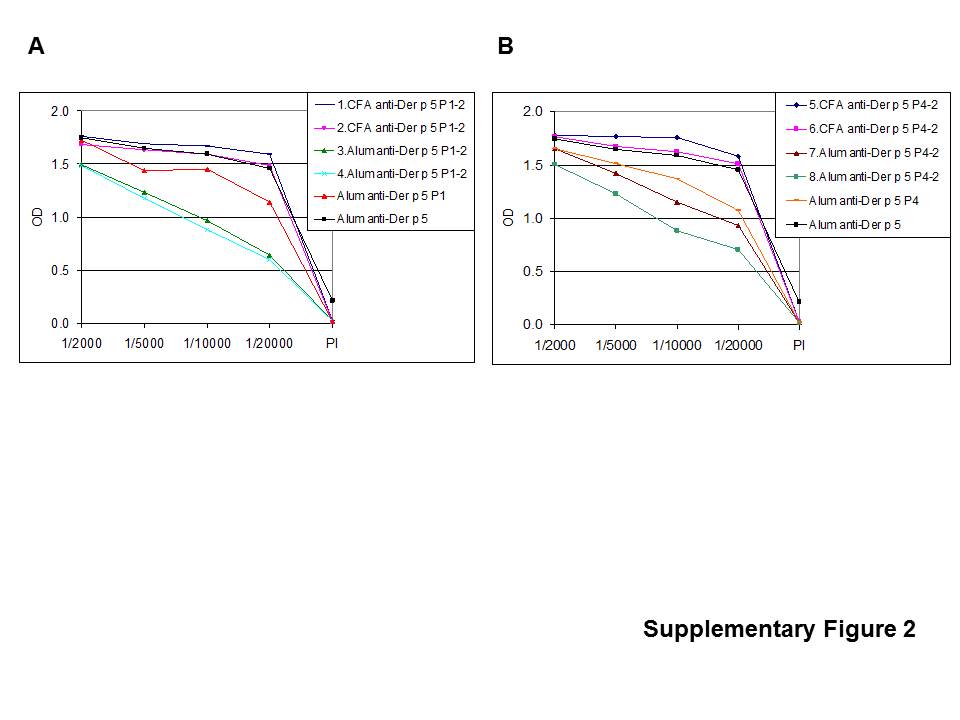

Supplement: Supplementary file 2 [file ALL-73-1653-s002.JPG]

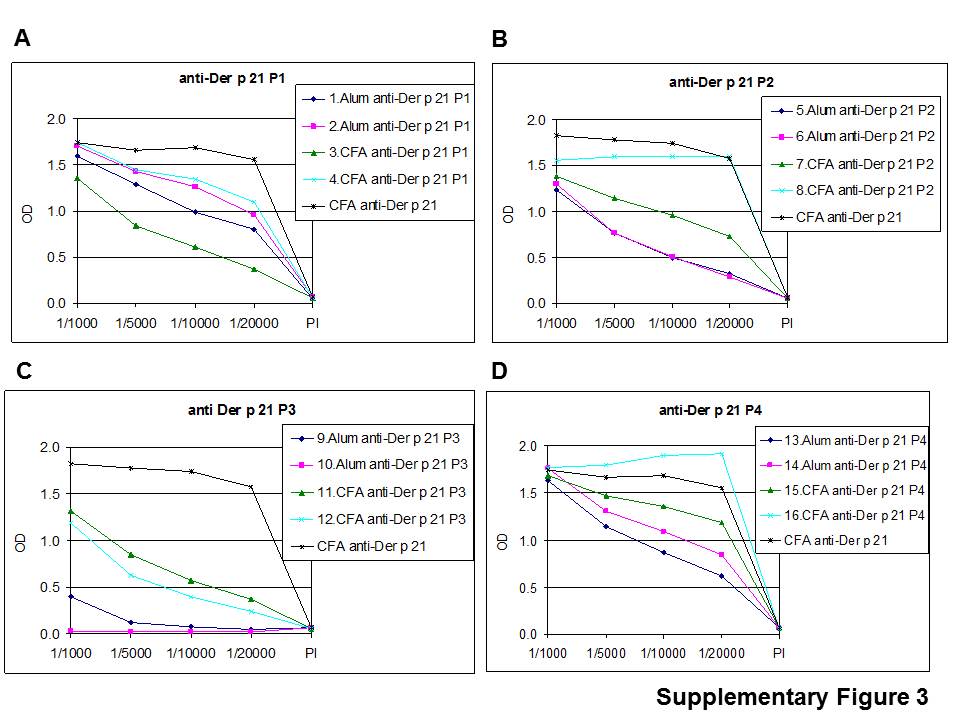

Supplement: Supplementary file 3 [file ALL-73-1653-s003.JPG]

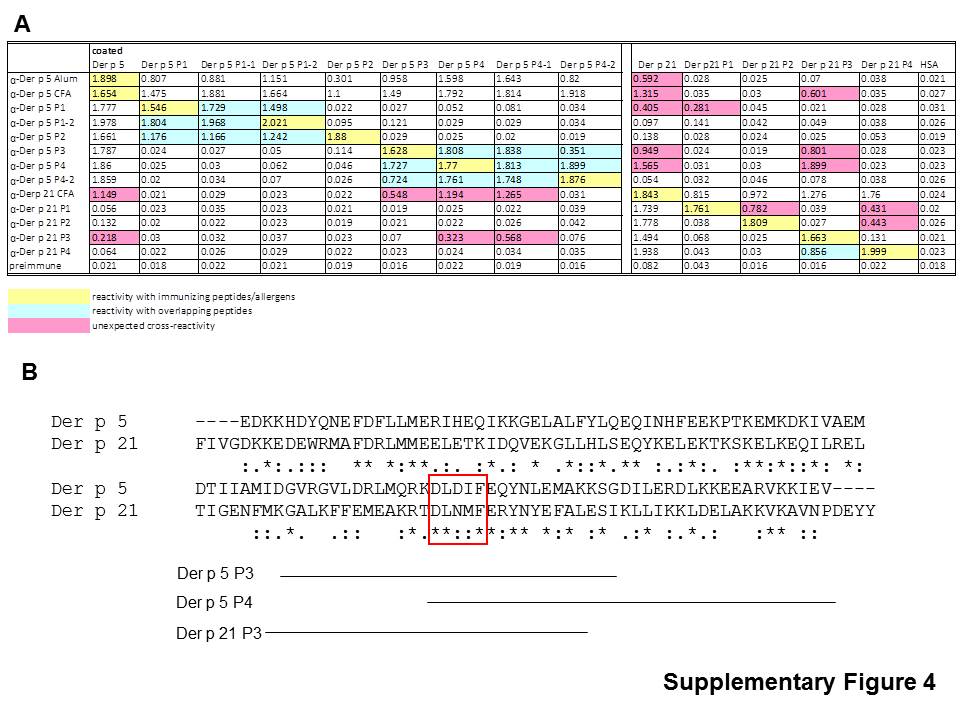

Supplement: Supplementary file 4 [file ALL-73-1653-s004.JPG]

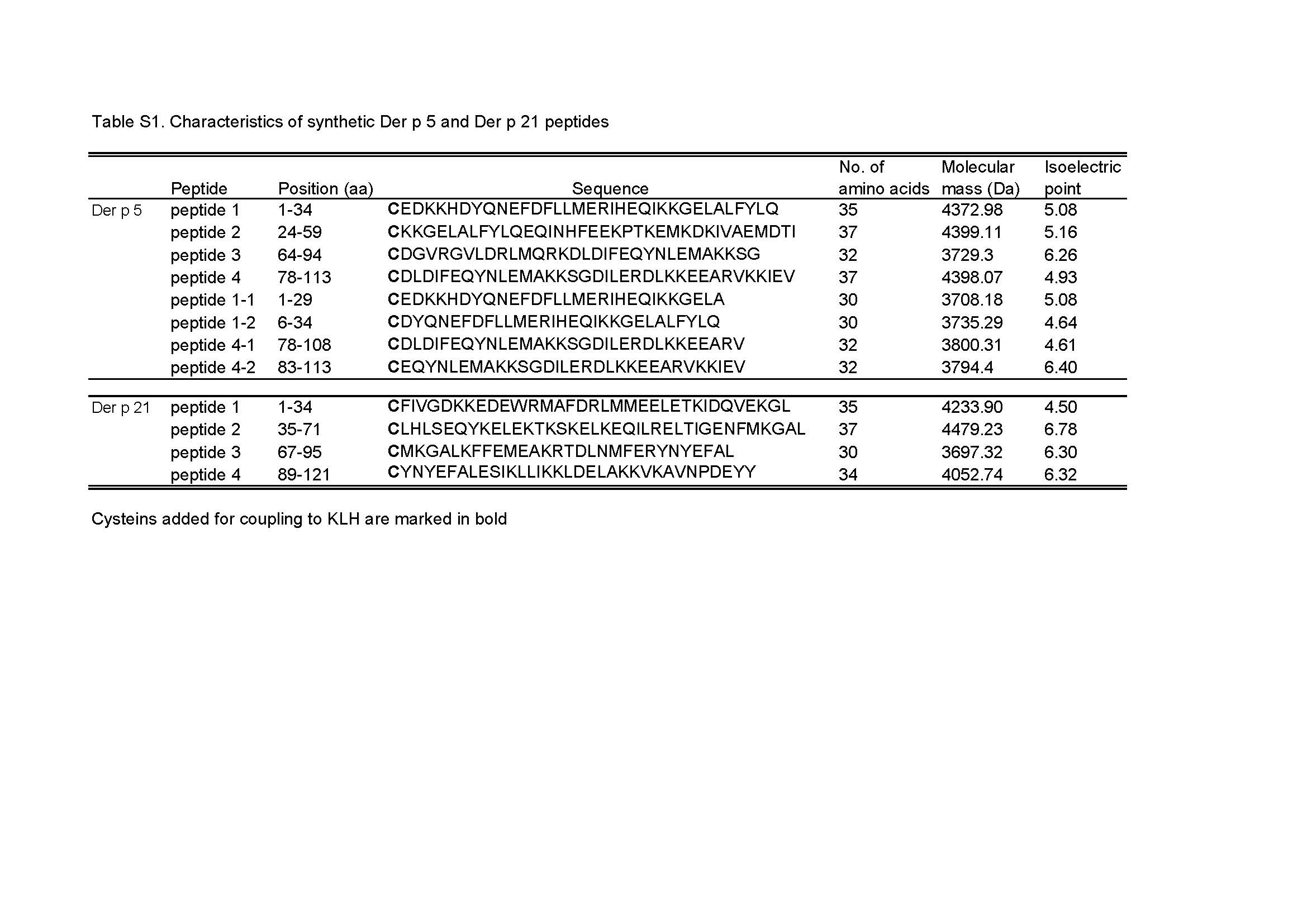

Supplement: Supplementary file 5 [file ALL-73-1653-s005.jpg]

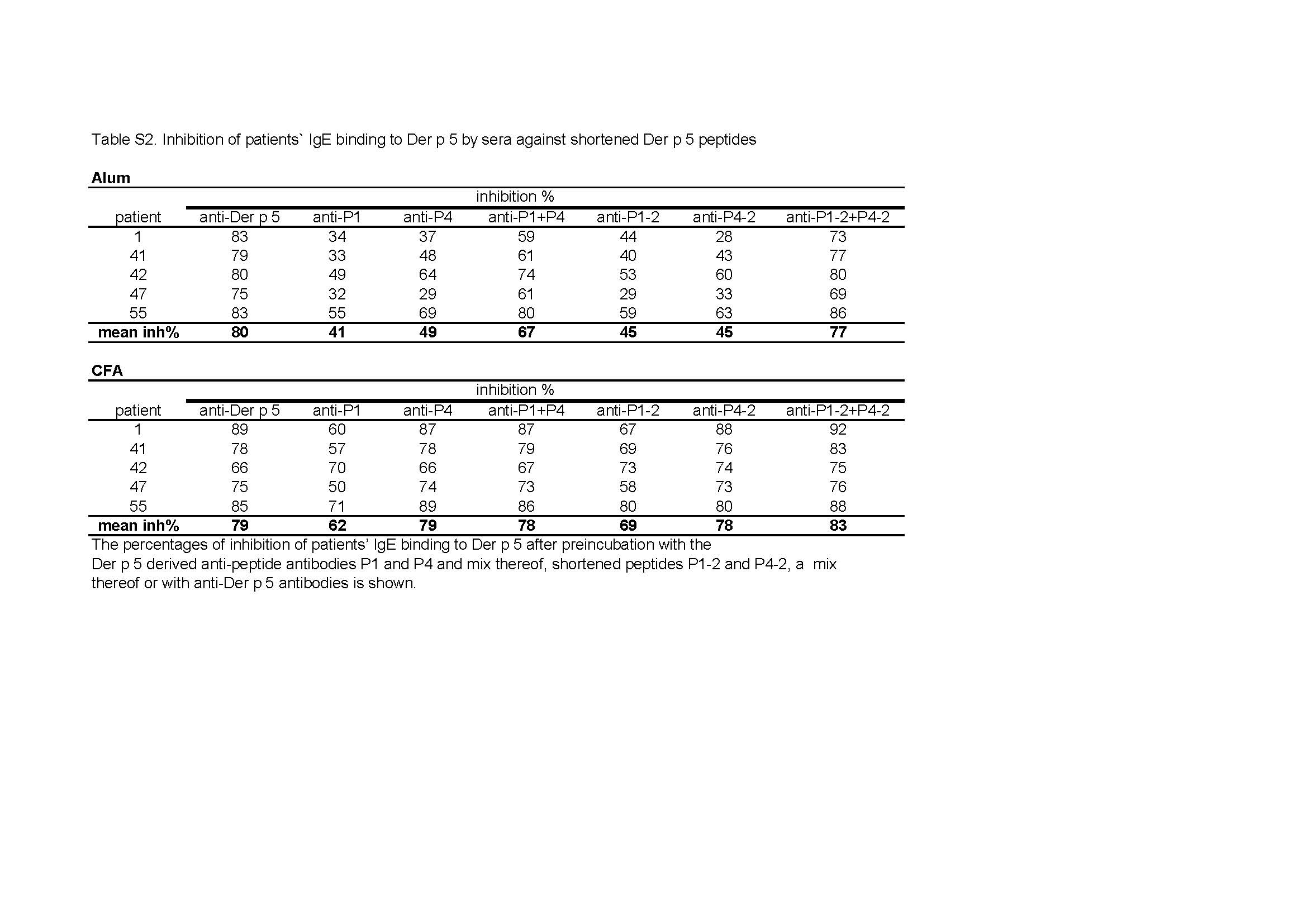

Supplement: Supplementary file 6 [file ALL-73-1653-s006.jpg]
