# Supplementary material for: Similar localization of conformational IgE epitopes on the house dust mite allergens Der p 5 and Der p 21 despite limited IgE cross‐reactivity
Source: Allergy. 2018 Feb 21;73(8):1653–61. doi: 10.1111/all.13398 (PMC6055609; doi:10.1111/all.13398)
Supplement: Supplementary file 7 [file ALL-73-1653-s007.docx]

**SUPPORTING INFORMATION**

***Sera from allergic patients***

Sera from HDM-allergic patients (n =55) were obtained from two allergy outpatient clinics and were selected according to the positive case history and serological analysis. All the patients exhibited IgE antibodies to House dust mite extract (d1) as measured by ImmunoCAP (Phadia, Thermofisher, Uppsala, Sweden) and IgE antibodies to Der p 5 and/or Der p 21 as determined by dot-blot. A blood sample from a non-allergic subject was used as a negative control. Blood and serum samples were analysed in an anonymous manner after the study was approved by the local ethics committee, Medical University of Vienna, Austria (EK 641/2014).

***Immunization of rabbits,*** ***determination of IgG antibody titres and testing for IgG cross-reactivity***

Peptide-specific IgG antibodies were obtained by immunizing rabbits three times (first booster injection after 4 weeks and a second booster injection after 7 weeks) with each of the KLH-conjugated peptides (200 µg/injection) and, for control purposes, with recombinant Der p 5 and Der p 21 protein (200 µg/injection) (Charles River, Chatillon sur Chalaronnne, France). In total four rabbits were immunized per peptide/protein, two using Freund’s adjuvant (1x complete and 2x incomplete Freund’s adjuvant) and two rabbits using aluminium hydroxide as adjuvant (SERVA Electrophoresis, Heidelberg, Germany). Pre-immune sera were obtained from the rabbits before immunization. Rabbit immune responses were analysed by ELISA titrations. For the measurement of specific rabbit IgG antibodies, ELISA plates (Nunc, Roskilde, Denmark) were coated overnight at 4°C with 2µg/mL of rDer p 5 or rDer p 21. After blocking with 1%BSA-TBST (10 mM Tris, 150 mM NaCl, 0.5% [v/v] Tween-20, PH 8) for 2.5h at 37°C, the plates were incubated overnight at 4°C with serial dilutions of the corresponding rabbit antisera, or the corresponding pre-immune sera (1 : 1.000, 1 : 5.000, 1 : 10.000 and 1 : 20.000 in 0.5%BSA-TBST). Bound rabbit IgG antibodies were detected with a 1 : 2000 diluted horseradish peroxidase–labelled donkey anti-rabbit IgG antiserum (Amersham Biosciences, Little Chalfont, UK). For testing cross-reactivity between Der p 5 and Der p 21 ELISA plates were coated with Der p5, Der p 21, Der p 5-derived peptides and Der p 21-derived peptides (2µg/mL). After blocking, plates were incubated with anti-Der p 5 antiserum, anti-Der p 21 antiserum, anti-Der p 5 peptide antisera, anti-Der p 21 peptide antisera, and with corresponding preimmune sera as a control. Bound rabbit IgG antibodies were detected as described above.

***IgE reactivity of dot-blotted Der p 5, Der p 21 and allergen-derived peptides***

Aliquots containing 0.5 µg of purified rDer p 5, Der p 5-derived peptides, rDer p 21, Der p 21-derived peptides and for control purpose BSA were dotted onto nitrocellulose membrane (Schleicher & Schuell, Dassel, Germany). Membranes were blocked with gold buffer (50 mM sodium phosphate [pH 7.4], 0.5% [v/v] Tween-20, 0.5% [w/v] BSA, and 0.05% [w/v] sodium azide), three times for 20 min and then incubated with HDM-allergic patients’ sera (diluted 1:10 in gold buffer) and with a serum from a non-allergic person (1:10 in gold buffer) overnight at 4°C. Bound IgE was detected with 1:10 diluted ^125^I-labeled anti-human IgE Abs (Demeditec Diagnostics, Kiel, Germany) and visualized by autoradiography (Kodak XOMAT film).

**SUPPLEMENTARY TABLES AND FIGURES:**

**TABLE S1.** Characteristics of Der p 5- and Der p 21-derived peptides

**TABLE S2.** Inhibition of patients IgE binding to Der p 5 with antisera specific for Der p 5 and shortened Der p 5 peptides.

**FIGURE S1.** Der p 5-derived peptides induce Der p 5-specific antibodies in rabbits. Rabbits were immunized with KLH-coupled Der p 5-derived peptides adsorbed to alum (2 rabbits per peptide) or to FA (2 rabbits per peptide). IgG reactivity of anti-sera to ELISA plate bound Der p 5 was determined for different dilutions (x-axes) (A: anti-Der p 5 P1, B: anti-Der p 5 P2, C: anti-Der p 5 P3, D: anti-Der p 5 P4) and compared with an anti-Der p 5 anti-serum and the corresponding pre-immune sera (PI). Bound IgG antibodies correspond to the optical density (OD) values (y-axes).

**FIGURE S2.** Comparison of the immunogenicity of Der p 5-derived peptides P1 and P4 with shortened peptides. Rabbits were immunized with KLH-coupled shortened Der p 5-derived peptides adsorbed to alum (2 rabbits per peptide) or to FA (2 rabbits per peptide). IgG reactivity of anti-sera to ELISA plate bound Der p 5 was determined for different dilutions (x-axes) (**A**: anti-Der p 5 P1-2, B: anti-Der p 5 P4-2) and compared with an anti-Der p 5 anti-serum raised with Alum-adsorbed Der p 5, and antisera raised against Alum-adsorbed KLH-P1 (A), KLH-P4 (B) and the corresponding pre-immune sera (PI)). Bound IgG antibodies correspond to the optical density (OD) values (y-axes).

**FIGURE S3.** Der p 21-derived peptides induce Der p 21-specific antibodies in rabbits. Rabbits were immunized with KLH-coupled Der p 21-derived peptides adsorbed to alum (2 rabbits per peptide) or to FA (2 rabbits per peptide). IgG reactivity of anti-sera to ELISA plate bound Der p 21 was determined for different dilutions (x-axes) (A: anti-Der p 21 P1, B: anti-Der p 21 P2, C: anti-Der p 21 P3, D: anti-Der p 21 P4) and compared with an anti-Der p 21 anti-serum. PI: corresponding pre-immune sera. Bound IgG antibodies correspond to the optical density (OD) values (y-axes).

**FIGURE S4.** Cross-reactivity of allergen-specific and peptide-specific rabbit anti-sera with Der p 5, Der p 21 and peptides. A, Allergens and peptides (top horizontal line) were coated on the ELISA plates and probed with the anti-sera and the corresponding pre-immune sera (left column). Bound IgG antibodies correspond to optical density (OD) values (means of duplicates; deviation<5%). Reactivity with the original immunogens, overlapping peptide sequences and cross-reactivities with small sequence motifs are indicated in yellow, blue and pink, respectively. B, Amino acid sequence alignment of Der p 5 and Der p 21. ***** identical residues, **:** amino acids with strong similarity, **.** amino acids with weak similarities. One motif responsible for IgG cross-reactivity between Der p 5 and Der p 21 is boxed in red. Positions of Der p 5 P3, Der p 5 P4 and Der p 21 P3 are underlined.
